# Supplementary material for: Islr regulates canonical Wnt signaling-mediated skeletal muscle regeneration by stabilizing Dishevelled-2 and preventing autophagy
Source: Nat Commun. 2018 Dec 3;9:5129. doi: 10.1038/s41467-018-07638-4 (PMC6277414; doi:10.1038/s41467-018-07638-4)
Supplement: Supplementary file 1 — Supplementary Information [file 41467_2018_7638_MOESM1_ESM.pdf]

Zhang et al., Islr regulates canonical Wnt signaling-mediated skeletal muscle regeneration by stabilizing Dishevelled-2 and preventing autophagy

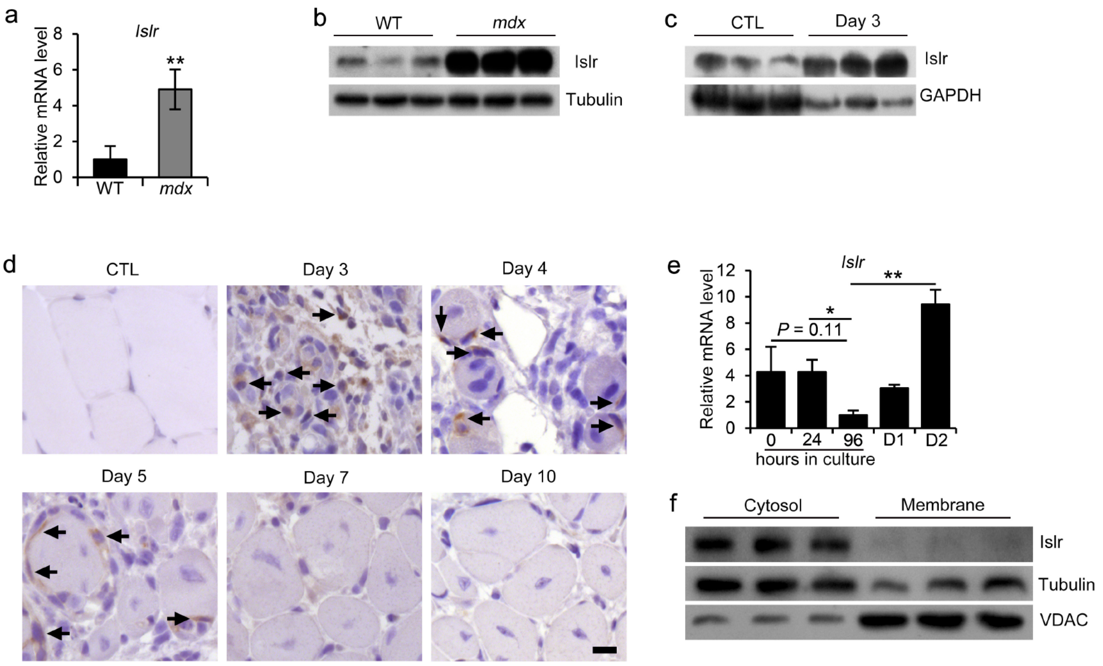

Supplemental Figure 1. Islr participates in adult skeletal muscle regeneration and satellite cell differentiation. (**a**) Expression analysis of *Islr* in wild type (WT) and *mdx* mice at the age of 9 months using quantitative real-time PCR (qRT-PCR). $N = 3$ in each group. (**b**) Western blot analysis of Islr protein levels in WT and *mdx* mice at the age of 9 months. $N = 3$ in each group. (**c**) Western blot analysis of Islr protein levels in injured and contralateral TA muscles (CTL) at 3 d post injury. (**d**) Immunohistochemistry analysis of Islr in injured TA muscles of WT mice at 3, 4, 5, 7, and 10 d post injury. Scale bar = 50 µm. (**e**) Expression analysis of *Islr* in freshly isolated satellite cells, activated satellite cells cultured for 24 h in proliferation medium, proliferating cells cultured for 96 h in proliferation medium, differentiating cells cultured for 4 d in proliferation medium followed by 1 d in differentiation medium (D1), and differentiating cells cultured for 4 d in proliferation medium followed by 2 d in differentiation medium (D2) using qRT-PCR. (**f)** Western blot analysis of Islr in cytosol and membrane fractions of differentiating cells cultured for 3 d in differentiation medium. Error bars represent the means ± s.d. $^{*}P < 0.05$, $^{**}P < 0.01$; Student's *t* test.

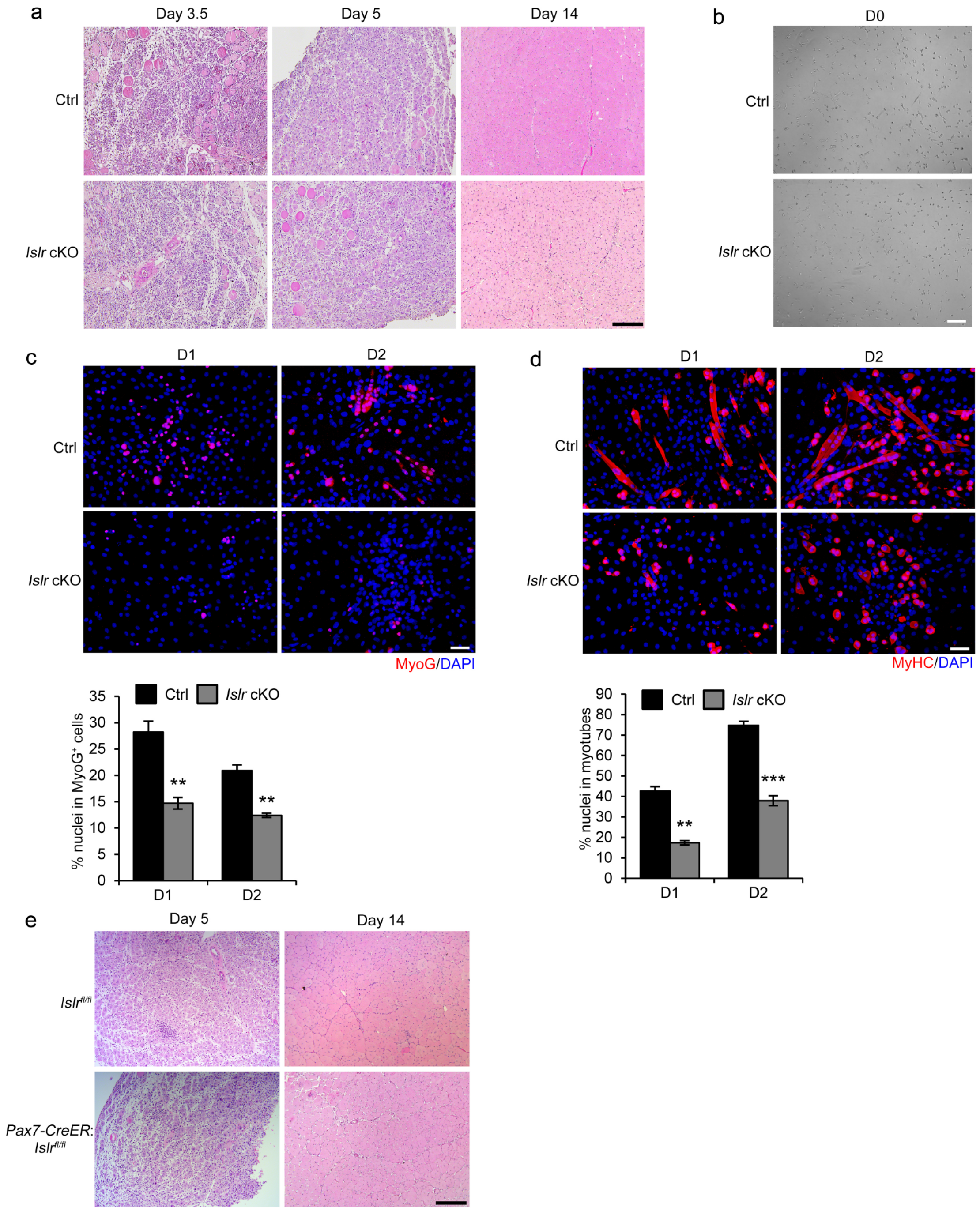

Supplemental Figure 2. Islr-deficiency delayed the progression of myogenesis. (**a**) Low magnification photos of **Fig. 2e**. N = 3 in each group. Scale bar = 200 μm. (**b**) Photos of isolated satellite cells from control and *Islr* cKO mice cultured for 4 d in proliferation medium before starting differentiation. N = 3 cell cultures in each group. Scale bar = 250 μm. (**c**) Immunofluorescence analysis of MyoG^{+} cells in primary myoblasts from control and *Islr* cKO mice after 1 (N = 3) and 2 (N = 3) d in differentiation medium (D1, D2). Scale bar = 50 μm. The percentages of MyoG^{+} cells are shown on the

below. (**d**) Immunofluorescence analysis of $MyHC^+$ cells in primary myoblasts from control and *Islr* cKO mice after 1 ($N = 3$) and 2 ($N = 3$) d in differentiation medium. Scale bar = 50 μm. The percentages of nuclei contained in the myotubes (a $MyHC^+$ cell with at least 2 nuclei) are shown on the right. (**e**) Low magnification photos of **Fig. 5d**. $N = 3$ in each group. Scale bar = 200 μm. Error bars represent the means ± s.d. $^{**}P < 0.01$, $^{***}P < 0.001$; Student's *t* test.

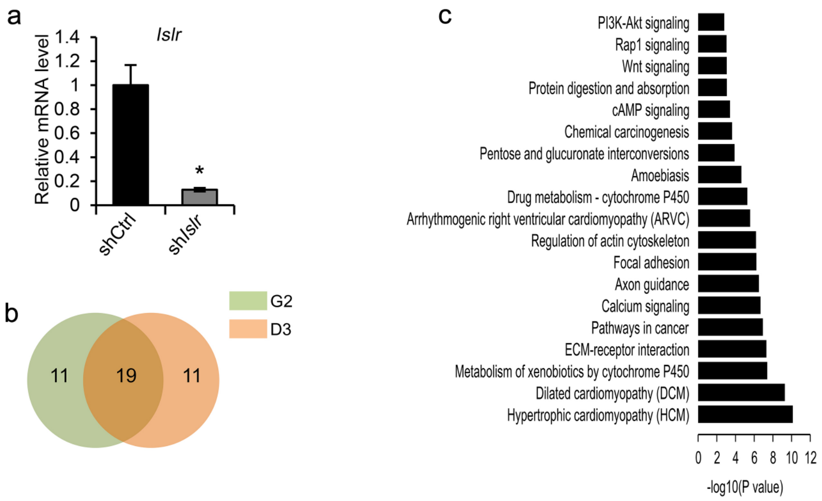

Supplemental Figure 3. Gene expression analysis of sh*Islr* C2C12 cells by RNA sequencing (RNA-seq). (**a**) Expression analysis of *Islr* in control shRNA (shCtrl) and *Islr* shRNA stable (sh*Islr*) C2C12 cells after 2 d in growth medium (G2) using qRT-PCR. (**b**) Gene pathways enriched in sh*Islr* C2C12 cells relative to shCtrl C2C12 cells at both G2 and D3. N = 3 cell cultures in each group. (**c**) The significance of 19 pathways in sh*Islr* C2C12 cells relative to shCtrl C2C12 cells at D3. N = 3 cell cultures in each group. Error bars represent the means ± s.d. $^{*}P < 0.05$; Student's t test.

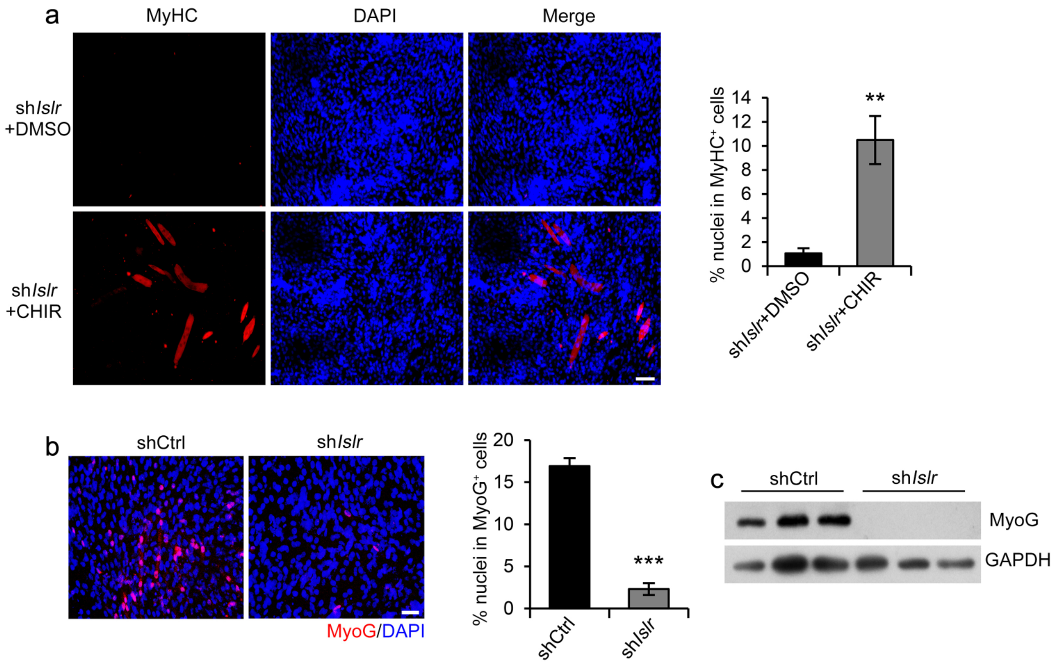

Supplemental Figure 4. CHIR rescues the failure of sh*Islr* C2C12 cells to differentiate, and the numbers of MyoG^{+} cells are decreased in sh*Islr* C2C12 cells. (**a**) Immunofluorescence staining for MyHC in sh*Islr* C2C12 cells treated with CHIR or DMSO after 3 d in differentiation medium. $N = 3$ cell cultures in each group. Scale bar = 100 μm. The percentages of MyHC^{+} cells are shown on the right. (**b**) Immunofluorescence staining for MyoG in shCtrl and sh*Islr* C2C12 cells after 3 d in differentiation medium. $N = 3$ cell cultures in each group. The percentages of MyoG^{+} cells are shown on the right. Scale bar = 50 μm. (**c**) Western blot analysis of MyoG protein levels in shCtrl and sh*Islr* C2C12 cells after 3 d in differentiation medium. Error bars represent the means ± s.d. $^{**}P < 0.01$, $^{***}P < 0.001$; Student's *t* test.

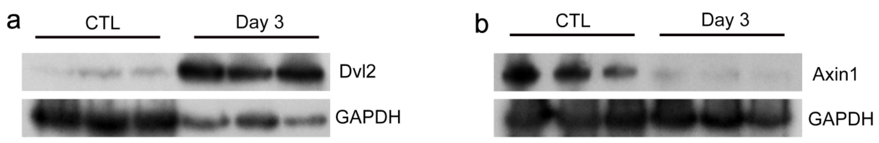

Supplemental Figure 5. The canonical Wnt signaling pathway is activated during skeletal muscle regeneration. (**a**) Western blot analysis of Dvl2 protein levels in injured and contralateral TA muscles at 3 d post injury. (**b**) Western blot analysis of Axin1 protein levels in injured and contralateral TA muscles at 3 d post injury.

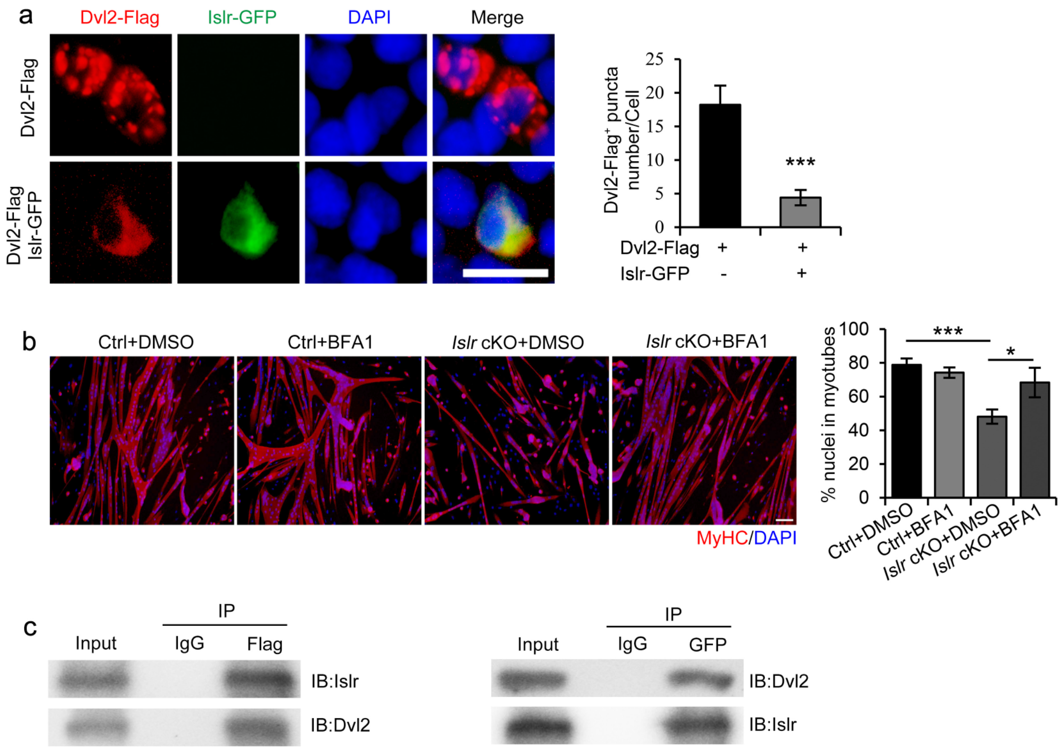

Supplemental Figure 6. Islr interacts with Dvl2 in HEK293T cells, and BFA1 rescued the phenotype of primary myoblasts of *Islr* cKO mice. (**a**) Immunofluorescence analysis of Dvl2-Flag^{+} puncta in HEK293T cells transfected with Dvl2-Flag plasmid or Dvl2-Flag and Islr-GFP plasmids. N = 3 cell cultures in each group. Approximately 20 cells total in each group. Scale bar = 25 µm. The numbers of Dvl2-Flag^{+} puncta per cell are shown on the right. (**b**) Immunofluorescence staining for MyHC in primary myoblasts of control and *Islr* cKO mice treated with BFA1 or DMSO for 24 h after 3 d in differentiation medium. N = 3 cell cultures in each group. Scale bar = 100 µm. The percentages of nuclei contained in the myotubes (a MyHC^{+} cell with at least 2 nuclei) are shown on the right. (**c**) Reciprocal co-immunoprecipitation analysis between GFP-tagged Islr and Flag-tagged Dvl2 in HEK293T cells. IB: immunoblotting; IP: immunoprecipitation. Error bars represent the means ± s.d. $^{*}P < 0.05$, $^{***}P < 0.001$; Student's t test.

Figure 1e

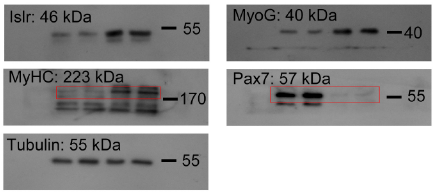

Figure 1k

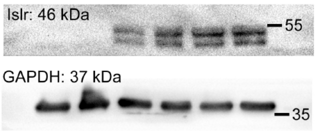

Figure 2b

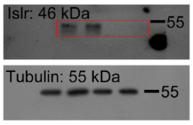

Figure 2d

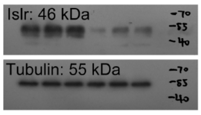

Figure 3c

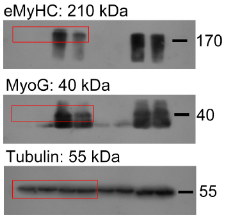

Figure 3d

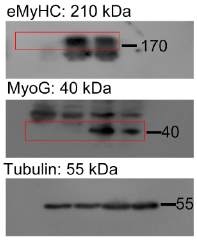

Figure 4c

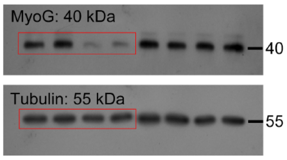

Figure 4f

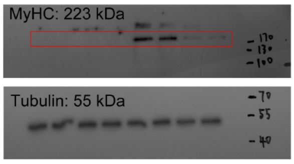

Figure 5c

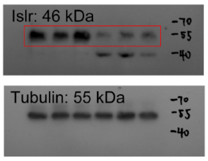

Figure 6e

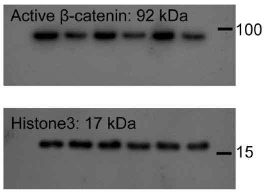

Figure 6g

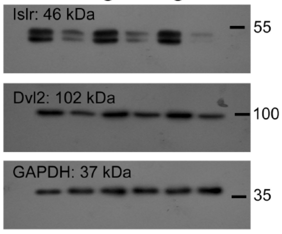

Figure 6h

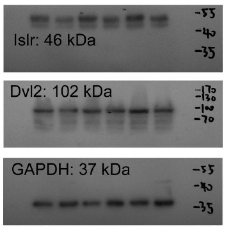

Figure 6i

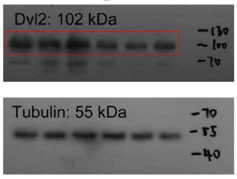

Figure 7a

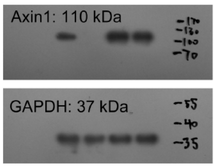

Figure 7b

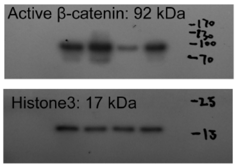

Figure 7c

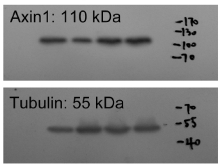

Figure 7d

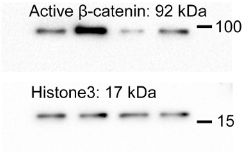

Figure 7e

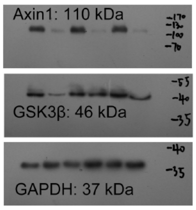

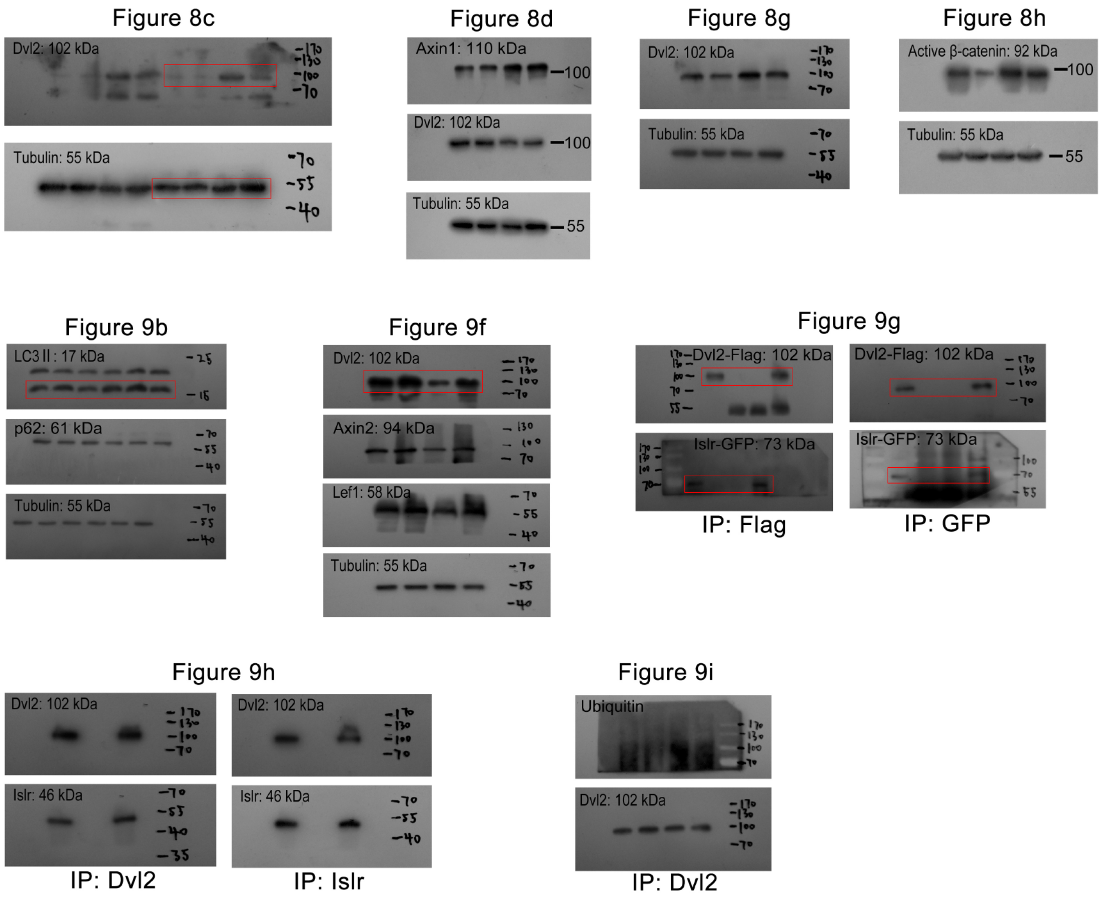

Supplemental Figure 7. Full scans of immunoblots shown in Figs 1-9.
